# Luteolin selectively kills STAT3 highly activated gastric cancer cells through enhancing the binding of STAT3 to SHP-1

**DOI:** 10.1038/cddis.2017.38

**Published:** 2017-02-09

**Authors:** Shiyu Song, Zhonglan Su, Hui Xu, Mengyuan Niu, Xiufang Chen, Haiyan Min, Bin Zhang, Guibo Sun, Sijing Xie, Hongwei Wang, Qian Gao

**Affiliations:** 1 Center for Translational Medicine and Jiangsu Key Laboratory of Molecular Medicine, Medical School of Nanjing University, Nanjing, China; 2 Department of Dermatology, The First Affiliated Hospital of Nanjing Medical University, Nanjing, China; 3 Department of Biochemistry, School of Basic Medical Sciences, Wenzhou Medical University, Wenzhou, China; 4 Central Laboratory, Nanjing Chest Hospital, Medical School of Southeast University, Nanjing, China; 5 Institute of Medicinal Plant Development, Chinese Academy of Medical Sciences and Peking Union Medical College, Beijing,China

## Abstract

The antitumor effect of luteolin, a plant flavonoid, in gastric cancer (GC) cells has not been fully understood. Here we show that luteolin selectively kills STAT3 overactivated GC cells that are often drug resistant. The treatment of luteolin in these GC cells significantly inhibited STAT3 phosphorylation and reduced the expression of STAT3 targeting gene Mcl-1, Survivin and Bcl-xl. Silencing of SHP-1, a protein tyrosine phosphatase, abolished the inhibitory effect of luteolin on STAT3 and cell apoptosis, suggesting that SHP-1 is crucial in luteolin-mediated cellular function. Moreover, this luteolin effect of STAT3 dephosphorylation by SHP-1 involved in HSP-90, which protected STAT3 phosphorylation by forming HSP-90/STAT3 complex. Thus, luteolin inhibited STAT3 activation through disrupting the binding of HSP-90 to STAT3, which promoted its interaction to SHP-1, resulted in the dephosphorylation of STAT3. The GC cell xenograft mouse model confirmed the effectiveness of luteolin induced inhibition of tumor growth *in vivo*.

Gastric cancer (GC) remains as a major public health concern worldwide. It is the fourth most common cancer and the second leading cause of cancer mortality with >700 000 deaths annually.^1^ The epidemiology data indicate that the countries in Eastern Asia, for example, China, Japan, Korea and Mongolia, are the high-risk regions for GC.^2^ Other countries, such as the countries in Eastern Europe and the part of Central and South America, also have high incidence rate of this malignancy.^3^

Signal transducer and activator of transcription 3 (STAT3) is a key transcription factor regulates cell growth, differentiation and survival.^4^ It can be activated by a variety of upstream signals, including cytokines, growth factors and oncogenes.^5^ In response to upstream signal stimuli, the recruited tyrosine kinase JAK induces STAT3 phosphorylation at tyrosine 705. The phosphorylated STAT3s then self-dimerize and shuttle to the nuclear, and regulate the transcription of its targeting genes. In the physiological conditions, STAT3 phosphorylation is tightly regulated and often transient. Multiple negative regulators, such as SOCS3, SHP-1 and PIAS, have crucial roles to keep STAT3 activity at low levels in normal conditions.^6, 7, 8, 9^ However, constitutive activation of STAT3 is frequently observed in tumor cells and is considered as ‘oncogenic’.^10, 11, 12, 13^ Clinically, the upregulation of STAT3 in cancer cells is linked with a worse prognosis, severer drug resistance and shorter survival period in patients.^14, 15^

Luteolin (3,4,5,7-tetrahydroxyflavone) is a natural flavonoid abundant in various fruits and vegetables. It usually occurs in its glycosylated form in celery, green pepper, perilla leaf and camomile tea.^16^ Luteolin has a wide spectrum of biological activities ranging from antioxidation to anti-inflammation and anti-allergy.^17, 18, 19^ In recent years, its anticancer effect has attracted attention.^17, 20, 21^ Various tumor cells have been found sensitive to luteolin treatment, including the cells from liver cancer, epidermal cancer, breast cancer and prostate cancer.^22, 23, 24, 25^ It was also found to be able to increase the radio or chemosensitivity of certain GC cell lines, but the mechanisms underlying were not known.^26, 27^

We have previously demonstrated that the inhibition of STAT3 could reverse the chemosensitivity of STAT3 overactivated GC cells. In the present study, we showed that these cells were also sensitive to luteolin, and a mechanism that involves SHP-1 and HSP-90, which explains how luteolin exerts its anticancer effect in STAT3 overactivated GC cells.

## Results

### Luteolin selectively induced apoptosis in drug-resistant GC cells

**Figure 1:**
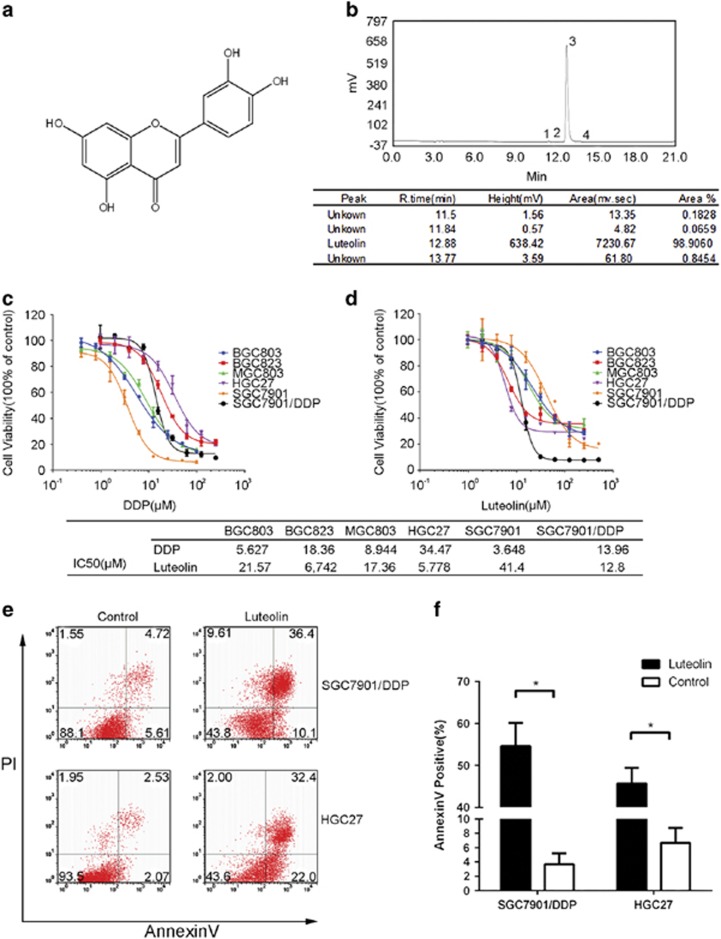
Luteolin selectively induced apoptosis in the drug-resistant GC cells. (**a**) Molecular structural formula of luteolin. (**b**) UV absorption measured by HPLC shown the purity of luteolin. (**c** and **d**) Various gastric tumor cells were treated with fold diluted DDP or luteolin. Cell viability was determined by a MTS method. IC_50_ value was determined to evaluate the inhibition of tumor cell growth by luteolin. The value of each cells was the mean value of three independent experiments. (**e**) SGC7901/DDP and HGC27 cells were treated with 15 or 5 *μ*M luteolin and then stained with propidium iodide and AnnexinV-FITC for detecting the apoptosis by flow cytometry. (**f**) The columns showed the statistical FACS result of three independent experiments, *P<0.05

The structural formula of luteolin and its purity were shown in Figures 1a and b. To test the antitumor effect of luteolin, a set of six GC cell lines was examined, including a cisplatin (DDP)-resistant cell line (SGC7901/DDP) initially derived from a Chinese GC cell line (SGC7901) and established by step-increasing DDP treatment.^28^ As shown in Figures 1c and d, we observed that the GC cells that were less sensitive to DPP had a higher sensitivity to luteolin. The effect of luteolin in SGC7901/DDP cells was several times higher than that of its parental SGC7901 cells (12.8 : 41.4 *μ*M, IC_50_). Similarly, the DDP-resistant GC cells, BGC823 and HGC27, were also sensitive to luteolin with the comparable low IC_50_ values as in SGC7901/DDP. To determine the nature of the cell death induced by luteolin, we performed a classical AnnexinV-PI double staining analysis and found that the treatment of luteolin had induced a massive cell apoptosis in the GC cell lines (Figures 1e and f), indicating that the cell death was likely the major reason for luteolin’s anti-GC cell effect.

### The antitumor effect of luteolin correlated with STAT3 inhibition

**Figure 2:**
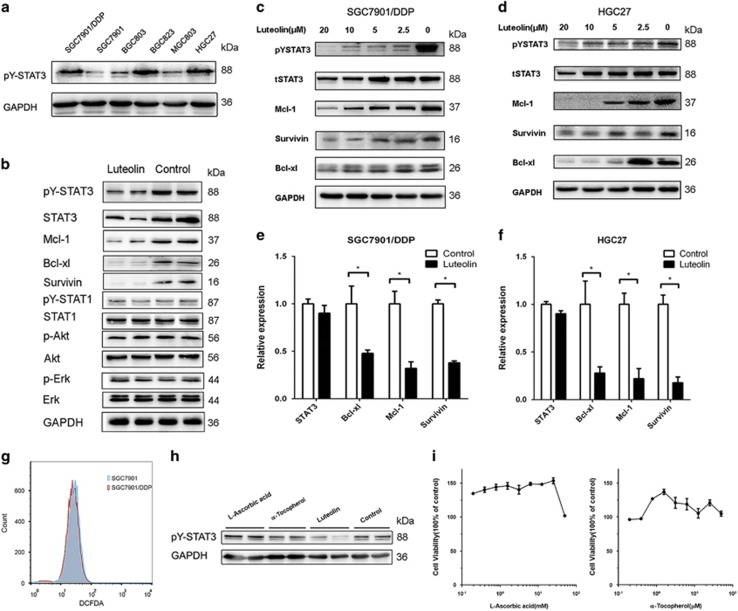
The antitumor effect of luteolin was correlated with STAT3 inhibition. (**a**) Different GC cell lines were subjected to western blot for measuring protein levels of phosphorylated STAT3. (**b**) The SGC7901/DDP cells were treated with 10 *μ*M luteolin and then were subjected to western blot for measuring protein levels by indicated antibodies. (**c** and **d**) SGC7901/DDP cells and HGC27 cells were treated with indicated concentrations of luteolin and then were subjected to western blot for measuring protein levels by indicated antibodies. (**e** and **f**) Total RNA of luteolin treated SGC7901/DDP and HGC27 tumor cells was extracted and a QPCR method was adopted to detect the transcription level of indicated genes. The result was obtained of three independent experiments. *P<0.05. (**g**) SGC7901 and SGC7901/DDP cells were incubated with DCFDA for 30 min, and the fluorescence levels were analyzed by flow cytometer. (**h**) Western blot analyses of pY-STAT3 level of antioxidants and luteolin treated SGC7901/DDP cells. (**i**) SGC7901/DDP tumor cells were treated with fold diluted drugs. Cell viability was determined by MTS

We previously observed that the high levels of STAT3 phosphorylation in SGC7901/DDP cells were causally related with their chemo drug resistance.^28^ In consistent, we found that all three drug-resistant GC cell lines, SGC7901/DDP, BGC823 and HGC27, exhibited higher activation of STAT3, when compared with those of drug-sensitive GC cell lines (Figure 2a). Thus, the antitumor effect of luteolin in GC cells may be through the inhibition of STAT3 signaling.

To evaluate this hypothesis, we initially questioned whether the antitumor effect of luteolin in GC cells was related to STAT3 function in SGC7901/DDP cells. As shown in Figure 2b, the treatment of luteolin (24 h) significantly inhibited STAT3 phosphorylation in SGC7901/DDP cells, whereas it did not show obvious effect on STAT1, Akt and Erk phosphorylation. This luteolin’ s effect was dose dependent confirmed in both SGC7901/DDP and HGC27 cells (Figures 2c and d). The expression levels of STAT3 downstream pro-survival proteins, Mcl-1, Bcl-xl and Survivin, were also significantly reduced. The fact that the mRNA levels of these genes were significantly downregulated upon luteolin treatment, confirming that the inhibition of these STAT3 downstream factors was at the transcriptional levels, likely through STAT3 inhibition (Figures 2e and f). As the reactive oxygen species (ROS) were known to activate STAT3 and promote tumor development.^29^ We then asked whether the antitumor effect of luteolin was depended on its antioxidant activity. To address this question, we evaluated the ROS levels of the drug-sensitive SGC7901 cells and drug-resistant SGC7901/DDP cells. As shown in Figure 2g, the ROS levels are equivalent and relatively low in these tumor cells. Furthermore, the hydrophilic antioxidant l-ascorbic acid and the hydrophobic antioxidant *α*-tocopherol could neither inhibit STAT3 phosphorylation nor induce cells death (Figures 2h and i), indicating that a specific mechanism other than luteolin’s anti-oxidative activity was involved.

### Luteolin inhibition of STAT3 is SHP-1 dependent

**Figure 3:**
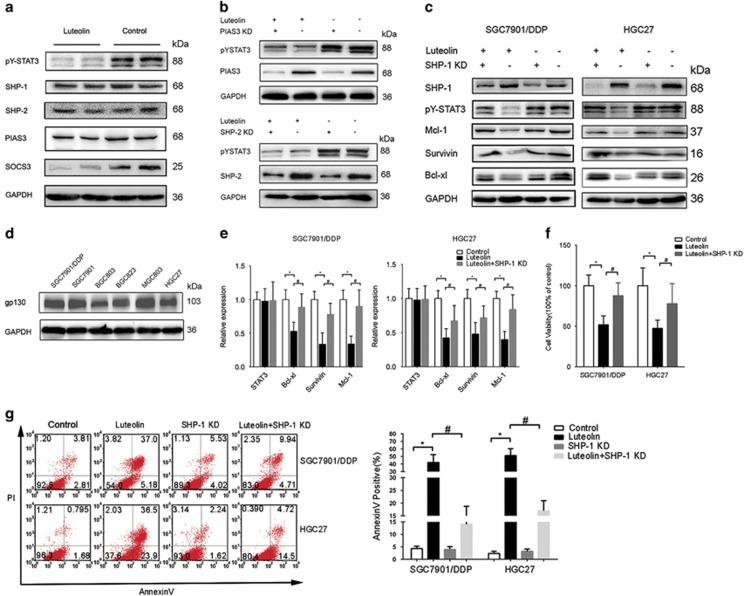
The inhibition of STAT3 by luteolin was SHP-1 dependent. (**a**) SGC7901/DDP cells treated with luteolin were subjected to western blot for measuring protein levels by indicated antibodies. (**b**) SGC7901/DDP cells were transfected with PIAS3 and SHP-2 siRNA or control siRNA and then incubated with (+) or without (−) luteolin. Western blotting with indicated antibodies was performed. (**c**) SGC7901/DDP and HGC27 cells were transfected with SHP-1 siRNA or control siRNA and incubated with (+) or without (−) luteolin. Western blotting with indicated antibodies was performed, respectively. (**d**) Different gastric tumor cell lines were subjected to western blot for measuring protein levels of gp130. (**e**) The total RNA of above-treated cells was extracted, and a QPCR method was adopted to test the transcription level of indicated genes. The result was obtained of three independent experiments. *P<0.05. (**f**) The viability of above treated cells was determined by MTS method. The result was obtained of three independent experiments. *P<0.05. (**g**) The above-treated cells was stained with propidium iodide and AnnexinV-FITC for detecting the apoptosis by flow cytometry. The right panel shown the static result of three independent experiments.*P*<0.05 (compare with control), ^#^*P*<0.05 (compared with luteolin only group)

Next, we questioned how luteolin inhibition of STAT3 phosphorylation in STAT3 hyperactivated GC cells was achieved. First, we evaluated the levels of STAT3 inhibitor SHP-1, SHP-2, SOCS3 and PIAS3.^7, 14, 30, 31^ Among these proteins, none of them showed an apparent increase after luteolin treatment. Instead, we observed a significant downregulation of SOCS3, a canonic STAT3 downstream component involving a negative feedback regulation of STAT3 signaling, presumably due to the inhibition of STAT3 transcriptional activity (Figure 3a). Moreover, knockdown of SHP-2 and PIAS3 had no influences on the inhibition of STAT3 phosphorylation by luteolin (Figure 3b), suggesting that these STAT3-negative regulators were not involved in luteolin’s effect on STAT3. In addition, the IL-6 receptor family partner gp130 levels were also not related to STAT3 activation in these tumor cell lines (Figure 3d).

On the other hand, after SHP-1 knockdown, the inhibition of STAT3 phosphorylation and its transcriptional activity by luteolin were largely attenuated, suggesting that the effect of luteolin on STAT3 is at least in part, if not all, SHP-1 dependent (Figures 3c and e). Furthermore, knockdown of SHP-1 also reduced the proapoptotic effect of luteolin, which was confirmed by MTS and FACS assay (Figures 3f and g).

### Luteolin disrupted the binding of HSP-90 to STAT3

**Figure 4:**
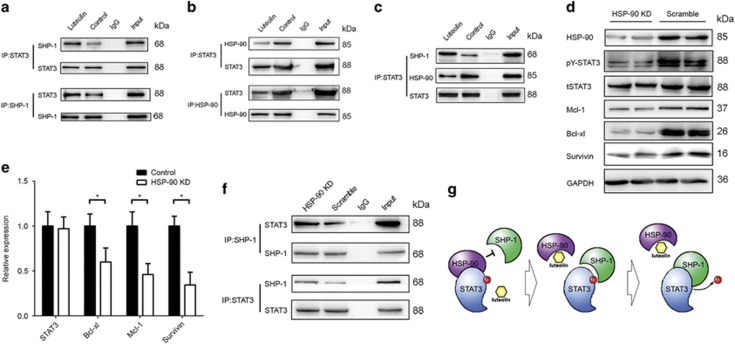
Luteolin disrupted the binding of HSP-90 to STAT3. (**a**) Lysates of SGC7901/DDP cells treated with luteolin or control were immunoprecipitated with anti-STAT3 antibody or control rabbit IgG, and the immunopellets were detected by immunoblot analysis with anti-SHP-1 and anti-STAT3 antibody. Cell lysates were then immunoprecipitated with anti-SHP-1 antibody or control rabbit IgG, and the immunopellets were detected by immunoblot analysis with the anti-STAT3 antibody and anti-SHP-1 antibody. (**b**) The lysates of above-treated cells were immunoprecipitated and blotted with indicated antibodies. (**c**) The lysates of luteolin-treated cells were immunoprecipitated with STAT3 antibody and blotted with SHP-1 and HSP-90 antibodies at same time. (**d**) SGC7901/DDP cells were transfected with HSP-90 siRNA or controlled siRNA. Western blotting with indicated antibodies was performed. (**e**) Total RNA of the above transfected cells of the cells was extracted and a QPCR method was adopted to test the transcription level of indicated genes. The result was obtained of three independent experiments, *P<0.05. (**f**) SGC7901/DDP cells were transfected with HSP-90 siRNA or control siRNA and then the cells were immunoprecipitated and blotted with indicated antibodies. (**g**) The model of the luteolin regulation of STAT3 phosphorylation

As the addition of luteolin did not alter the expression of SHP-1, we hypothesized that the SHP-1-dependent effect of luteolin on STAT3 is through the promotion of the binding of SHP-1 to STAT3. To test this hypothesis, a co-Ip assay was performed. As shown in Figure 4a, the treatment of luteolin, indeed, enhanced the binding of SHP-1 to STAT3. It was previously shown that a non-coding RNA lnc-DC is capable of disrupting SHP-1 to STAT3 binding in dendritic cells,^32^ raised the possibility that the treatment of luteolin may have changed lnc-DC content in luteolin treated cells. However, in our context, we did not observe any regulation of lnc-DC by luteolin in tested GC cells (data not shown). Earlier, we observed that HSP-90 is involved in the STAT3 phosphorylation (unpublished data) and Luo’s group showed that luteolin could bind to HSP-90 directly, and decrease STAT3 phosphorylation.^33^ To investigate whether HSP-90 had a role in the context of STAT3 hyperactivation, we immunoprecipitated either HSP-90 or STAT3 and blotted STAT3, SHP-1 or HSP-90, respectively. As shown in Figures 4b and c, either HSP-90 or SHP-1 precipitated with STAT3, and luteolin altered the binding pattern of STAT3 to HSP-90 and SHP-1 simultaneously in an opposite manner. The binding of STAT3 to either HSP-90 or SHP-1 were mutually exclusive. And no direct binding between SHP-1 to HSP-90 was observed. Finally, knockdown of HSP-90 mimicked the effect of luteolin on STAT3 phosphorylation and its downstream gene expression at both protein (Figure 4d) and transcriptional (Figure 4e) levels, which facilitated the binding of SHP-1 to STAT3 (Figure 4f). Thus, luteolin reduced the levels of STAT3 phosphorylation by disrupting the binding of HSP-90 to STAT3 (Figure 4g).

### Luteolin inhibited gastric tumor growth *in vivo*

**Figure 5:**
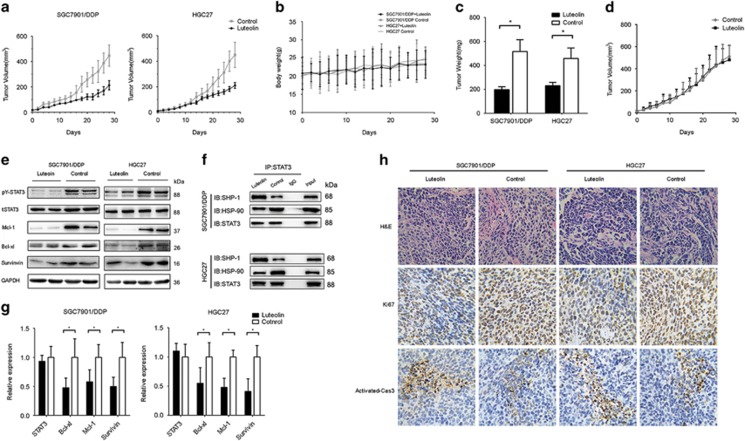
Luteolin inhibited gastric tumor growth *in vivo*. SGC7901, SGC7901/DDP and HGC27 cells were injected into male BALB/c nude mice (*N*=10). After tumors grew to about 100 mm^3^, mice were treated with or without luteolin (20 mg/kg/day), intraperitoneally. (**a**) Tumor volumes of SGC7901/DDP and HGC27 baring mice were calculated every other day. (**b**) The weight of each mouse was measured every other day before the treatment of drugs. (**c**) The tumor was removed and weighted at the end of the experiment. *P<0.05. (**d**) Tumor volume of SGC7901 baring mice was calculated every other day. (**e**) Lysates from tumor tissue were analyzed by western blotting and probed with indicated antibodies. (**f**) The tumor issues were lysed and an Ip assay was performed by indicated antibodies. (**g**) mRNA levels of indicated genes from tumor tissue was tested by QPCR. *P<0.05. (**h**) Histopathology of xenograft tumors stained with H&E, anti-Ki-67 antibody and anti-activated caspase-3 antibodies. Original magnification 100-fold

To assay whether the effect of luteolin in GC cells may be clinically relevant, we tested the effect of luteolin on tumor growth *in vivo*. The xenograft mouse models with different GC cells were established by subcutaneous injections of drug-resistant SGC7901/DDP, HGC27 cells or non-drug-resistant SGC7901 cells, respectively. When the tumors reached 100 mm^3^ in volume, the mice received either vehicle (PBS) or luteolin (20 mg/kg/day) for 4 weeks. Both SGC7901/DDP and HGC27 bearing mice (*n*=10) that received luteolin showed a growth inhibition of the tumors (Figure 5a), but not the body weight, when compared with those of control groups (*n*=10, Figure 5b), suggesting a high efficacy and low toxicity of luteolin *in vivo*. The average tumor weights in luteolin treatment SGC7901/DDP and HGC27 bearing mice were 61.6% or 49.5% of those of controls, respectively (Figure 5c). In consistent with our cellular results, we did not find the differences in the tumor volumes between luteolin or vehicle-treated non-drug-resistant SGC7901 bearing mice (*n*=10, Figure 5d).

Next, we measure the levels of STAT3 phosphorylation in tumor samples and found that the *in vivo* treatment of luteolin significantly downregulated STAT3 phosphorylation and Mcl-1, Bcl-xl, Survivin expression at both protein and transcription levels (Figures 5e and g). Furthermore, we observed that luteolin disrupted the binding of HSP-90 to STAT3 and enhanced the binding of SHP-1 to STAT3 *in vivo* (Figure 5f). Immunohistochemistry showed that the cell proliferation marker Ki-67 was downregulated, whereas the levels of activated caspase-3 was significantly upregulated in the luteolin-treated mice bearing STAT3 highly activated GC cells (Figure 5h). Together, our data indicated that luteolin inhibited drug-resistant GC cell growth both *in vitro* and *in vivo* through a STAT3 inhibition mechanism involving SHP-1 and HSP-90.

## Discussion

STAT3 is a key transcription factor involving in inflammation, angiogenesis, wound healing, metabolism and proliferation. It is considered as an oncogene for its tumor-promoting effect. Various methods targeting STAT3 in tumor treatment showed beneficial effects in both preclinical and clinical studies.^34, 35, 36^ However, tumor cells are highly heterogenic, sharing very different levels of STAT3 activity. The mechanisms that cause hyperactivity of STAT3 in different cells vary significantly. They may result from the lack of negative feedback regulations, over-activation of kinases, dysfunction of phosphatases or expansion of upstream receptors.^5^ It is, therefore, important to elucidate the specific mechanisms underlying STAT3 activation in different tumor cells for precise tumor therapies.

As a flavonoid, luteolin has shown a potent anticancer activity in various tumor cells. However, the underlying mechanisms of luteolin effects remain largely unclear. Several unrelated signaling pathways were suggested to be inhibited by luteolin including Akt-Gsk-cyclin D pathway in nasopharyngeal carcinoma, PKC and c-Src pathways in UVB-induced skin cancer and Nrf2 signaling in lung cancer.^22, 24, 25^ These findings raised the question whether luteolin indeed targets different pathways in different cells, or there is a common mechanism shared by various luteolin-sensitive cells. A recent finding showed that luteolin can inhibit tumor growth through STAT3 pathway.^37^ Our previous work demonstrated that the levels of STAT3 phosphorylation are associated with gastric tumor chemoresistance *in vitro*.^28^ Here we further tested the proapoptotic effect of luteolin on GC cells. We found that the cells with STAT3 hyperactivated were highly sensitive to luteolin, when compared with non-STAT3 hyperactivated cells. It is reported that luteolin may induce ubiquitin depended protein degradation, affecting phosphorylated STAT3 in hepatoma cells, which may also influence the levels of total STAT3.^38^ However, we failed to identify this effect of luteolin in the GC cells as a dominate function (data not shown). And in some conditions, ROS may promote tumor development by inducing STAT3 activation through src, JAK or NF-κB signaling, and this effect may be reversed by anti-oxidative reagents.^39, 40, 41^ Yet, we did not observe any positive effect of antioxidant on STAT3 activation. Instead, we found that the knockdown of SHP-1 significantly disrupted the anticancer effect of luteolin in STAT3 highly activated cells tested. Importantly, the expression of SHP-1 was not regulated by luteolin. Further study uncovered that the treatment of luteolin increased the binding of SHP-1 to STAT3 and promoted STAT3 dephosphorylation.

The balanced action of protein tyrosine kinases and protein tyrosine phosphatases (PTPs) is crucial in keeping tyrosine phosphorylation levels at a dynamic equilibrium in biological systems. SHP-1 (also designated as SHPTP-1, SHP, HCP and PTPIC) is mainly restricted to hematopoietic and epithelial cells.^31, 42^ It is widely accepted as a negative regulator of tyrosine phosphorylation signaling pathways. Although extensive studies on SHP-1 have revealed that the expression of SHP-1 was compromised in most, if not all, cancer cell lines and tumor tissues examined.^13, 43, 44^ For example, compound like acetyl-11-keto-B-boswellic acid was able to inhibit constitutive STAT3 activation in multiple human myeloma cell lines by upregulating the expression of SHP-1.^43^ However, in our study, the regulation of SHP-1 function by luteolin is not at protein expression level. Nevertheless, knockdown of SHP-1 attenuated the effect of luteolin on STAT3. Previously, it was suggested that the binding of HSP-90 to STAT3 promotes STAT3 phosphorylation. Here, we found that luteolin reduced the binding of HSP-90 to STAT3, released phosphorylated STAT3 from HSP-90 chaperone, which may cause phosphor-STAT3 exposed to both phosphatases and proteases, followed by the declining of STAT3 phosphorylation, as a dominant phenomenon, and in some circumstances total STAT3.

Finally, the *in vivo* study recaptured our findings *in vitro*, that is, luteolin has a strong antitumor effect on the xenograft animals established by injections of STAT3 highly activated, but not STAT3 poorly activated, GC cells with a low toxicity. STAT3 phosphorylation was strongly inhibited in these xenografts treated by luteolin and the target genes of STAT3 were also downregulated at both transcription and protein levels. Thus, the effect of luteolin is highly selective to STAT3 ‘addicted’ tumor cells. It was suggested that STAT3 hyperactivated tumor cells may have a vital role in tumor development, featuring as tumor stem cells.^45, 46^ Selectively targeting this subpopulation of cells may have a great influence on tumor growth.

In conclusion, we have investigated the capability of luteolin in inhibition of STAT3 hyperactivated GC cells and the possible mechanism underlying. We uncovered a competitive binding mechanism of HSP-90 or SHP-1 to STAT3, which regulates the balance of STAT3 activation in cells. This novel finding may be adopted to the treatment of STAT3 addicted tumors.

## Materials and methods

### Reagents and antibodies

Luteolin is purified and provided from Key Laboratory of Bioactive Substances and Resources Utilization of Chinese Herbal Medicine (Beijing, China), and the purity of the product was over 98%, detected by HPLC (UV). Gastric tumor cell lines of SGC7901, SGC7901/DDP, HGC27, MGC803, BGC803 and BGC823 were purchased from Keygene (Jiangsu, China). Monoclonal antibodies against HSP-90, STAT3 and phosphor-STAT3 (Tyr705), STAT1 and phosphor-STAT1 (Tyr701), Akt, phosphor-Akt (Ser473), Erk and Phospho-p44/42 MAPK (Erk1/2) (Thr202/Tyr204) were purchased from Cell Signaling Technology (Boston, MA, USA). Monoclonal antibodies against SHP-1, SHP-2, Bcl-xl, Survivin, Mcl-1, SOCS3, PIAS3, gp130, ki0–67, activated caspase-3 and GAPDH were purchased from Abcam (Burlingame, CA, USA). BCA Protein Assay Kit was purchased from Pierce (Rockford, IL, USA). l-Ascorbic acid was purchased from Cayman (Ann Arbor, MI, USA), *α*-Tocopherol was purchased from Selleckchem (Houston, TX, USA). DCFDA was purchased from Sigma-Aldrich (St. Louis, MO, USA).

### Cell culture and transfection

The above-mentioned cells are maintained in RPMI-1640 medium (Life Technology, New York, NY, USA) supplemented with 10% fetal bovine serum (Hyclone, Logan, UT, USA), 100 units/ml penicillin and 100 mg/ml streptomycin (Hyclone), in humidified 5% CO_2_ at 37 °C. The transfection of siRNA was performed using the Lipofectamine RNAimax transfection reagent (Life Technology) following the manufacturer’s guidelines. Briefly, tumor cells were seeded in a six-well plate at 60–80% confluence, 5 pmol of siRNA was diluted with opt-MEM media and mixed with transfection reagent each well. After transfection for 24 h, the cells were challenged with different treatment. siRNA sequences: SHP-1 (5′-GCACCAUCAUCCACCUUAA-3′, nonsense: 5′-ACUUACCGCAAUUCCAACC-3′), HSP-90 (5′-GUCAAGCUUUCAUACCGGAUU-3′, nonsense: 5′-AUCAUGGCUAUGUUGAACCUC-3′), SHP-2 (5′-AAUUCUAUAAAAUAUAAUGUU-3′, nonsense: 5′-AUAUUAAUCGUAAUAUUAUAA-3′), PIAS3 (5′-AAUGAUAAGAGAUUCAUAGGG-3′, nonsense: 5′-AGAAGGAUAUCGGAGUAAUAU-3′).

### Cell viability and apoptosis assay

Cells were seeded in triplicates at a density of 1 × 10^5^ cells/ml in 96-well plate, and cell viability assays were performed using the CellTiter 96 AQueous One Solution Cell Proliferation Assay kit (Promega, Madison, WI, USA). After treatment with twofold diluted drugs for 24 h, the absorbance at 490 nm was measured using a Microplate reader (Bioteck, Winooski, VT, USA). For apoptosis assays, cells cultured in a six-well plate were harvested and stained with AnnexinV-FITC and propidium iodide and assessed for the percentage of AnnexinV-positive population with a Calibur flow cytometer (BD, Franklin Lake, NJ, USA), and the data were analyzed with FlowJo Version 7.6.2 software (TreeStar, Ashland, OR, USA).

### Cell ROS detection

The ROS level of SGC7901 and SGC7901/DDP was detected by DCFDA with FACS according to manufacturer’s instructions. Briefly, the cells seeded in six-well plates were labeled with 20 *μ*M DCFDA at 37 °C for 30 min in the dark. And then the cells were collected and analyzed at FITC channel with flow cytometer.

### Immunoblotting and immunoprecipitation

Cells and tissues for immunoblotting were lysed by RIPA buffer on ice, and then the concentration of protein was detected by a BCA protein kit. In all, 50 *μ*g protein of each sample was resolved on 10% sodium dodecyl sulfate polyacrylamide gel electrophoresis, transferred to nitrocellulose membrane and detected by indicated antibodies. Cells for immunoprecipitation assay were lysed by a WB/IP buffer and proteins were immunoprecipitated with indicated antibodies, respectively. The precleared Protein A/G Plus-Agarose beads (Thermo, Waltham, MA, USA) were incubated with immunocomplexes overnight and washed three times with lysis buffer. The immunoprecipitates were subjected to SDS-PAGE followed by immunoblotting assay.

### Real-time PCR

Total mRNA was extracted from cultured cells and tumor sphere using RNeasy Micro Kit (Qiagen, Hilden, Germany), mRNA was reverse transcripted into cDNA with PrimeScript RT Master mix (TaKaRa, Otsu, Japan). SYBR green quantitative real-time PCR was performed, using PCR Master Mix (Life Technology). The relative expression of target genes was determined to beta-actin and was calculated by ΔΔCt method. Primers: STAT3: forward: 5′-CTGCTCCAGGTACCGTGTGT-3′, reverse: 5′-CCTCTGCCGGAGAAACAG-3′ Mcl-1, forward: 5′-GGCTAAACACTTGAAGACCATAA-3′, reverse: 5′-GAAGAACTCCACAAACCCATC-3′ Bcl-xl, forward: 5′-AAAGCGTAGACAAGGAGATGC-3′, reverse: 5′-TCCCATAGAGTTCCACAAAAGT-3′ Survivin, forward: 5′-TTACGCCTGTAATACCAGCAC-3′, reverse: 5′-TCACCAAGGGTTAATTCTTCA-3′ beta-actin, forward: 5′-GGACGACATGGAGAAAATCTG-3′, reverse: 5′-GGTCTCAAACATGATCTGGGT-3′.

### Mouse xenograft assays

Six-week-male Nude Balb/c mice were obtained from Model Animal Research Center of Nanjing University, cultured in a SPF condition. Mouse care and *in vivo* experimental procedures were approved by the Institutional Animal Care and Use Committee of the Nanjing University. A cell suspension of SGC7901, SGC7901/DDP an HGC27 cell (2 × 10^6^) in PBS was injected subcutaneously into nude mice’s right flank region. About 10 days later of the injection, the tumor cells formed measurable tumor sphere. And then the mice were divided randomly into different groups (*N*=10), receiving different treatment. Tumor-bearing mice were treated with luteolin (20 mg/kg) or PBS as control by intraperitoneal injection every 2 days. The volumes of the tumor were measured before each treatment. At the end of the experiment, mice were killed and the tumor spheres were removed by surgery and weighted to evaluate the inhibition of the drug.

### Immunohistochemistry paraffin

Immunohistochemistry was performed by a standard protocol. Briefly, the tumor spheres were removed from implanted region and fix with 4% paraformaldehyde and embedded in paraffin. After hydrolysis and antigen retrieval, the slides of both tumor baring mouse and human patients were blocked and washed with PBS. Immunostaining was carried out with rabbit monoclonal antibody to Ki-67 and activated caspase-3 at 4 °C overnight, respectively. And an UltraVision Quanto Detection System (Thermo) was adopted to detect the expression of indicated proteins.

### Statistical analysis

Mouse and tumor size, weights for each group were compared using Student’s *t*-test (for comparisons of two groups) and analysis of variance (for multiple group comparisons). For cell-based assays, samples with three replications were tested and calculated. For values that were not normally distributed (as determined by the Kolmogrov–Smirnov test), the Mann–Whitney’s rank sum test was used. A *P*-value <0.05 was deemed statistically significant. All statistical tests were two-sided and were performed using Graphpad prism 6 (GraphPad Software, La Jolla, CA, USA).
