# Correction: Luteolin selectively kills STAT3 highly activated gastric cancer cells through enhancing the binding of STAT3 to SHP-1

**DOI:** 10.1038/s41419-018-0827-z

**Published:** 2018-07-16

**Authors:** Shiyu Song, Zhonglan Su, Hui Xu, Mengyuan Niu, Xiufang Chen, Haiyan Min, Bin Zhang, Guibo Sun, Sijing Xie, Hongwei Wang, Qian Gao

**Affiliations:** 1 Center for Translational Medicine and Jiangsu Key Laboratory of Molecular Medicine, Medical School of Nanjing University, Nanjing, China; 2 Department of Dermatology, The First Affiliated Hospital of Nanjing Medical University, Nanjing, China; 3 Department of Biochemistry, School of Basic Medical Sciences, Wenzhou Medical University, Wenzhou, China; 4 Central Laboratory, Nanjing Chest Hospital, Medical School of Southeast University, Nanjing, China; 5 Institute of Medicinal Plant Development, Chinese Academy of Medical Sciences and Peking Union Medical College, Beijing, China

Correction to: *Cell Death & Dieases.*; 10.1038/cddis.2017.38; published online 9 February 2017.

The PDF and HTML versions of the article have been updated to include the Creative Commons Attribution 4.0 International License information.

